# Social Media Use and HIV Screening Uptake Among Deaf Adults in the United States: Cross-Sectional Survey Study

**DOI:** 10.2196/13658

**Published:** 2019-10-02

**Authors:** Michael Argenyi, Poorna Kushalnagar

**Affiliations:** 1 Gallaudet University Washington, DC United States; 2 University of Massachusetts Medical School Worchester, MA United States

**Keywords:** HIV, sexually transmitted disease, sexually transmitted infection, deaf, sign language, social media, internet

## Abstract

**Background:**

About 46% of US adults obtain recommended HIV screening at least once during their lifetime. There is little knowledge of screening rates among deaf and hard-of-hearing adults who primarily use American Sign Language (ASL), or of social media as a potentially efficacious route for HIV prevention outreach, despite lower HIV/AIDS-specific health literacy and potentially higher HIV seropositivity rates than hearing peers.

**Objective:**

We investigated both the likelihood of HIV screening uptake among deaf adults in the past year and over one year ago, and the relationship between social media use and HIV screening uptake among deaf adult ASL users.

**Methods:**

The Health Information National Trends Survey in ASL was administered to 1340 deaf US adults between 2015-2018. Modified Poisson with robust standard errors was used to assess the relationship between social media usage as a predictor and HIV screening as an outcome (screened more than one year ago, screened within the past year, and never been screened), after adjusting for sociodemographics and sexually transmitted disease (STD) covariates.

**Results:**

The estimated lifetime prevalence of HIV screening uptake among our sample was 54% (719/1340), with 32% (429/1340) in the past year. Being of younger age, male gender, black, lesbian, gay, bisexual, or queer, or having some college education or a prior STD were associated with HIV screening uptake. Adjusting for correlates, social media use was significantly associated with HIV screening in the past year, compared to either lifetime or never.

**Conclusions:**

Screening falls well short of universal screening targets, with gaps among heterosexual, female, Caucasian, or older deaf adults. HIV screening outreach may not be effective because of technological or linguistic inaccessibility, rendering ASL users an underrecognized minority group. However, social media is still a powerful tool, particularly among younger deaf adults at risk for HIV.

## Introduction

The United States Department of Health and Human Services and the Centers for Disease Control and Prevention (CDC) prioritize HIV screening tests as a cornerstone of the national HIV prevention strategy [1,2]. The United States Preventive Services Task Force (USPSTF) recommends all adults 15-65 be screened for HIV at least once, with increased frequency of screening among men who have sex with men (MSM), injection drug users, and high-prevalence settings, including sexually transmitted disease (STD) clinics, homeless shelters, tuberculosis clinics, and correctional settings [3]. However, data from the Behavioral Risk Factor Surveillance System (BRFSS) shows that only 36.1% of all adults, and 46% of adults ages 18-64, have reported ever being tested for HIV [4,5]. Despite the USPSTF recommendation, many physicians screen based on risk stratification or by patient request [6-8]. Factors found to increase the likelihood that a person receives an HIV test include: being MSM [9,10], a young adult [11], black [10,12], having recent STD testing or an STD diagnosis [13], and having access to HIV screening [13]. Gender has been found to indicate both higher and lower likelihood of receiving HIV screening [9-11,13].

Scant data exists on HIV screening uptake among deaf American Sign Language (ASL) users, who represent a medically underrepresented linguistic and cultural group [14]. One ASL adaptation of the BRFSS survey given to 282 (mean age 44.6 years) deaf adults in Rochester, New York, reported a lifetime screening rate of 47.5% in 2008, though this sample reflected primarily Caucasian, higher-educated, deaf adults and did not include diverse members from other regions in the United States [15].

One strategy for increasing HIV screening uptake may be implemented through social networking sites. People increasingly use social networking sites (eg, Facebook, Twitter) and mobile networking sites (eg, Grindr, Tinder) as avenues for sexual health information [16,17]. Several studies reported that adolescents and young adults were most likely to use social media or the internet in general [17], including using them to seek health information [16,18]. One systematic review of studies on communication about HIV prevention and treatment via internet platforms identified online outreach benefits, which included: increased access to information, increased communication among users, and between consumers and professionals, anonymity, community, and geographical reach [19].

Perhaps because of these benefits, online health information seeking is particularly prevalent among gay and bisexual men compared to lesbian or bisexual women and heterosexual peers [20]. Higher rates of internet use were associated with increased screening among MSM in a 2014 cross-sectional study of 9613 MSM across 20 US cities [17]. Social media use was also linked to increased HIV screening, although to our knowledge all currently published studies target adolescents or MSM and transgender women adults. Among 42 black MSM aged 18-30 in Los Angeles, a Facebook-mediated video intervention resulted in seven times higher odds of subsequent HIV screening over six weeks postintervention [21]. Another study found that MSM individuals who discussed HIV prevention and treatment in a closed Facebook group requested an HIV test kit more often compared to MSM individuals who did not take part in online discussion [22]. Conversely, at least one study in Australia found that never-tested MSM were more likely to spend time on social networking websites [23].

Recent studies support that deaf adults use the Internet as readily as their peers. In a study of 515 deaf adults, those who engaged in social media networking were more likely to discuss health issues with their healthcare providers via electronic platforms compared to nonusers of social media sites, potentially reducing communication barriers that contribute to health disparities [24]. Similarly, deaf gay, bisexual, and queer (GBQ) men who connected with lesbian, gay, bisexual, transgender, and queer (LGBTQ) peers online were more likely to be aware of preexposure prophylaxis (PrEP) compared to deaf GBQ men who did not have online connections [25,26]. Belief in PrEP’s effectiveness was also associated with discussing LGBTQ issues online or through social media (odds ratio [OR] 3.12; 95% CI 1.12-8.75) [26].

To better understand the need for tailored and accessible HIV prevention and treatment services in the deaf community, this study utilized data from a US sample of deaf ASL users to investigate: (1) the sample rate of HIV screening uptake; and (2) the association of HIV screening uptake with social media use after adjusting for correlates.

## Methods

### Data Source

Data for this study was drawn from a large Health Information National Trends Survey in American Sign Language (HINTS-ASL) dataset [27]. This included HIV or STD items which were administered to US deaf adults from October 2015 to March 2016, and October 2016 to May 2018. Prior to survey administration, all items were translated into ASL, tested through cognitive interviews with deaf adults, and the final translations were captured on film [27]. All participants were either born or became deaf before the age of 13.

### Survey Items

For the purposes of the current study, sexual orientation was assessed with the question, “What is your sexual orientation?” with response options of gay, lesbian, heterosexual, bisexual, asexual, queer, and other, please specify. Gender was assessed with the question, “What gender do you identify as now?” with response options of male, female, and nonbinary or genderqueer.

STD history was assessed with the question, “Have you ever had a sexually transmitted disease? IF YES, once or more than once?” Response options were once, more than once, and never. A question about HIV screening was asked as follows: “When was the last time that you were tested for HIV?” Response options were less than 3 months ago, in the past year, more than 1 year ago, and I have never been tested for HIV. A binary response was used for the social media question: “In the last 12 months, have you used the Internet to visit a social networking site, such as Facebook or Twitter?” Finally, regular healthcare provider was measured using the binary question: “Not including psychiatrists and other mental health professionals, is there a particular doctor, nurse, or other health professional that you see most often?”

### Procedure

Following Institutional Review Board approval by Gallaudet University, research staff recruited deaf ASL users throughout the United States. Recruitment methods included snowball sampling through personal networks [28], distribution of flyers, and advertisements on deaf-centered organizations’ websites and electronic newsletters. Communication between the research staff and participants occurred through accessible channels, including mail, email, social media, and video chat programs. Only those who self-reported using ASL as their primary language were included because this group was identified as a medically underserved group [29-31], while exclusion criteria included being under the age of 18 years old or having unilateral hearing loss. Deaf lesbian, gay, bisexual, and queer (LGBQ) individuals were oversampled to create a high-powered sample.

After the participant viewed the information in ASL and English online, the participant was directed to a page where they could choose to provide consent to participate or decline. Following consent, the fully accessible ASL-English online survey took approximately one hour to complete. Each participant received a $25 gift card for participating in the study. If the participant met with the research staff remotely, ASL instructions were given through a videoconferencing method and a URL survey link was emailed to the participant. The research staff remained visible to the participant through video conferencing and were readily available to answer questions or troubleshoot as the participant progressed through the consent document and survey. For on-site survey administration, the research staff stayed in the interview room with the participant. If the participant did not feel comfortable watching the ASL question as signed on the pre-recorded video, a research staff repeated and signed the question for the participant. For some participants, such as those with low vision or who did not feel comfortable with self-administration on a computer, the research staff signed all the questions and response options and recorded the participants’ responses on the computer. No names or identifying information were included in the online survey and a unique identifier was used to avoid storing personal information in the same online survey dataset. The identifying information was stored in a separate database that was accessible only to the principal investigator.

### Statistical Analyses

Descriptive statistics, including chi-square and two-tailed *t* tests, were used to summarize the sample characteristics. The level of significance was set at *P*<.05. Unweighted descriptive statistics, such as cross-tabulation and percentage procedures, were used to describe the sample. Responses to the HIV screening uptake question were recoded into three groups: (1) had HIV screening within 1 year; (2) had HIV screening more than 1 year ago; and (3) had never been screened. Social networking site usage was entered as the main predictor, along with other sociodemographic variables, for HIV screening uptake. To evaluate the association of social media both in the short-term (within the past one year) and long-term (lifetime) with never being tested for HIV, we ran two separate modified Poisson analyses with robust standard errors using binary outcomes: (1) never been screened (referent group) versus screened within the past year; and (2) never been screened (referent) versus screened more than one year ago. Modified Poisson regression with robust standard errors was used to calculate prevalence ratio estimates and 95% confidence intervals for HIV screening uptake with social media usage as the main predictor, adjusting for sociodemographic and sexual health indicators. The modified Poisson approach was recommended for models with binomial outcomes, and the application of robust standard errors helps rectify the overestimation for the prevalence ratio of HIV screening uptake [32].

The adjusted prevalence ratios (aPR) were used to estimate the likelihood of being screened for HIV within the past year, or more than a year ago, compared to never been screened (reference category). Covariates included age, gender, race, education, sexual orientation, and STD history. Age groups were chosen as the commonly used 18-24, 25-34, 35-49, and 50 and older, with 50 and older being the referent group. Sexual orientation was dichotomized as heterosexual and LGBQ. STD history was dichotomized as any STD history or none. Due to the low-powered sample size for the nonbinary or genderqueer group, this gender identity subgroup was not entered in the modified Poisson model. SPSS version 25.0 (IBM Corp, Armonk) was used for all analyses.

## Results

A total of 1340 deaf adult ASL users (66%, 883/1340 Caucasian; 65%, 874/1340 heterosexual) who responded to the question about HIV screening met the criteria for inclusion in this analysis. Geographically, approximately 40% (536/1340) of the respondents were from the South, 30% (402/1340) were from the West, 17% (228/1340) were Midwestern, and 13% (174/1340) were from the Northeast. In our sample, the estimated lifetime prevalence of HIV screening was 54% (719/1340). Table 1 describes and compares the characteristics of 338 (25%) deaf participants who were screened for HIV within one year, 381/1340 (28%**)** deaf participants who received screening more than one year ago, and 621/1340 (46%) deaf participants who have never been screened. Table 1 includes the distribution of age, sex at birth, present gender identity, sexual orientation, and race of our diverse sample. Because STD history implied an opportunity for concurrent HIV screening, further analysis revealed 151/1322 (11%) people with a history of one STD and 65/1322 (5%) people with more than one STD.

Chi-square analyses showed significant group differences in HIV screening uptake rates for gender, age, race, education, sexual orientation, history of having an STD, and having visited a social networking site in the last 12 months. Table 2 shows the adjusted prevalence ratios of demographic and sexual health factors associated with HIV screening uptake, relative to the never screened category, from a modified Poisson with robust standard errors model.

**Table 1:** General sociodemographic characteristics by time of HIV screening uptake.

Variable		Overall (N=1340), n (%)	HIV screening uptake within one year (n=338), n (%)	HIV screening uptake more than one year (n=381), n (%)	Never had HIV screening (n=621), n (%)	χ^2^_df_	*P* value
**Age groups**						**135.57_6_**	**.001**
	18-24 years old	235 (18)	78 (33)	35 (15)	122 (52)		
	25-34 years old	335 (25)	118 (34)	107 (32)	110 (33)		
	35-49 years old	370 (27)	90 (24)	149 (40)	131 (35)		
	>50 years old	400 (30)	52 (13)	90 (23)	258 (64)		
**Birth sex**						**22.60_2_**	**.001**
	Male	545 (41)	174 (32)	135 (25)	236 (43)		
	Female	792 (59)	163 (21)	244 (31)	385 (49)		
**Gender identity**						**28.48_4_**	**.001**
	Male	544 (41)	167 (31)	134 (24)	243 (45)		
	Female	763 (57)	158 (21)	232 (30)	373 (49)		
	Nonbinary or genderqueer	30 (2)	12 (40)	13 (43)	5 (17)		
	Missing data^a^	3 (0)	1 (33)	2 (67)	0 (0)		
**Race**						**24.95_6_**	**.001**
	Causcasian	883 (66)	187 (21)	261 (30)	435 (49)		
	Black	130 (10)	43 (33)	42 (32)	45 (35)		
	Latinx	181 (14)	60 (33)	43 (24)	78 (43)		
	Other	139 (10)	44 (32)	35 (25)	60 (43)		
	Missing data	7 (0)	4 (57)	0 (0)	3 (43)		
**Education**						**68.13_4_**	**.001**
	High school	263 (20)	54 (21)	40 (15)	169 (64)		
	Some college	300 (22)	98 (33)	65 (22)	137 (46)		
	College graduate	776 (58)	185 (34)	276 (36)	315 (41)		
	Missing data	1 (0)	1 (100)	0 (0)	0 (0)		
**Sexual Orientation**						**91.56_4_**	**.001**
	Gay or lesbian	263 (20)	109 (41)	86 (33)	68 (26)		
	Heterosexual	874 (65)	170 (20)	227 (26)	477 (55)		
	Bisexual	181 (14)	58 (32)	63 (35)	60 (33)		
	Missing data	22 (1)	1 (5)	5 (23)	16 (73)		
**Have STD^b^**						**103.64_4_**	**.001**
	Once	151 (11)	58 (38)	66 (44)	27 (18)		
	More than once	65 (5)	32 (49)	27 (42)	6 (9)		
	Never	1106 (83)	242 (22)	285 (26)	579 (52)		
	Missing data	18 (1)	6 (33)	3 (17)	9 (50)		
**Regular provider**						**1.03_2_**	**.60**
	No	524 (39)	139 (27)	142 (27)	243 (46)		
	Yes	804 (60)	198 (25)	236 (29)	370 (46)		
	Missing data	12 (1)	1 (8)	3 (25)	8 (67)		

**Visited a social networking site in the last 12 months**						**18.36_2_**	**.001**
	No	75 (6)	7 (9)	20 (27)	48 (64)		
	Yes	797 (60)	231 (29)	239 (30)	327 (41)		
	Missing data	468 (34)	100 (21)	122 (26)	246 (53)		

^a^Participants were permitted to skip questions that they did not wish to answer.

^b^STD: sexually transmitted disease.

**Table 2:** Modified Poisson with robust standard errors estimation of adjusted prevalence ratios for HIV screening status.

Demographics		Screened within one year versus never screened, aPR^a^ (95% CI)	*P* value	Screened more than one year ago versus never screened, aPR (95% CI)	*P* value
**Age**					
	18-24	1.99 (1.32-3.01)	.001	0.90 (0.61-1.33)	.60
	25-34	2.25 (1.55-3.28)	<.001	1.48 (1.15-1.91)	.003
	35-49	2.07 (1.40-3.05)	<.001	1.64 (1.28-2.09)	<.001
	>50	Reference	—^b^	Reference	—
**Gender**					
	Female	Reference	—	Reference	—
	Male	1.25 (1.03-1.50)	.02	0.94 (0.78-1.12)	.47
**Race**					
	Caucasian	Reference	—	Reference	—
	Black	1.56 (1.18-2.05)	.002	1.33 (1.04-1.70)	.02
	Latinx	1.33 (1.05-1.68)	.02	0.99 (0.76-1.29)	.92
	Others	1.06 (0.83-1.35)	.67	0.91 (0.63-1.31)	.60
**Education**					
	High School	0.85 (0.59-1.21)	.36	0.53 (0.36-0.80)	.002
	Some College	1.12 (0.91-1.04)	.29	0.86 (0.68-1.10)	.23
	College	Reference	—	Reference	—
**Sexual orientation**					
	Heterosexual	Reference	—	Reference	—
	LGBQ^c^	1.73 (1.41-2.12)	<.001	1.54 (1.30-1.83)	<.001
**STD^d^**					
	Never tested	Reference	—	Reference	—
	Tested	1.76 (1.49-2.08)	<.001	1.77 (1.49-2.10)	<.001
**Social media use**					
	Never used	Reference	—	Reference	—
	Used	2.01 (1.09-3.70)	.03	0.97 (0.68-1.39)	.87

^a^aPR: adjusted prevalence ratios.

^b^Not applicable.

^c^LGBQ: lesbian, gay, bisexual, and queer.

^d^STD: sexually transmitted disease.

As shown in Table 2 for adjusted prevalence ratios, respondents who were younger were incrementally more likely to have had HIV screening within one year. With female gender as a reference group, those who reported male gender had higher relative prevalence of HIV screening uptake within the past year only. Compared with those with a college degree, people who had only high school education had lower prevalence of having had been tested for HIV more than one year ago. Similarly, those who self-identified as black or Latinx were significantly more likely to have been screened in the past year than those who self-identified as Caucasian. Self-identification as LGBQ and having a positive STD history were associated with prevalent HIV screening uptake. Social media use was positively associated with HIV screening uptake within the past year, but not more than one year ago.

## Discussion

### Key Results

Prior research has explored demographic characteristics associated with HIV screening uptake [9-13], as well as the potential of using the internet and social networking sites as outreach avenues to increase HIV screening, particularly among high risk groups [16,19,22,33,34]. Our study expands such research to US-based deaf adults who use ASL, estimates the prevalence of HIV screening uptake in this medically underserved group, and investigates the role of social media as a predictor of HIV screening uptake among deaf adults.

Overall, our sample showed a higher lifetime prevalence of HIV screening (54%) compared to other studies drawn from the US population [4,5]. This is consistent with the 2016 BRFSS survey data that showed people with disabilities had similar or higher rates of lifetime HIV screening [15,35,36], possibly reflecting that people with disabilities, including deaf adults, often have increased frequency of healthcare visits and higher likelihood of Medicaid-sponsored health insurance coverage. However, the lifetime screening rate of 54% in our sample is modest, far from recommended universal screening targets [37], and may be influenced by our oversampling of LGBQ individuals who typically get tested more often than nonLGBQ individuals.

Screening rates within the past twelve months or one year were low in our sample (25%) yet higher than a national sample (10.1%), likely for the same reason as lifetime screening rates [38]. Such low rates are concerning, because 75% of a nationally representative probability sample of adults (n=3174) reported sexual activity in the past year in the 2015 National Survey of Sexual Health and Behavior (NSSHB) [39]. Rates of sexual activity among deaf adults appears to be like hearing peers: only 18.4% of 282 deaf adult residents in Rochester, NY, reported abstinence in the past 12 months in the 2008 Deaf Health Survey, which is like 19.3% of 1890 hearing adults (ages 18 to 64) in the 2006 Adult Health Survey [15]. More recently, only 24.8% of a nationally representative probability sample of adults in the 2015 NSSHB survey reported not being sexually active in the past year [39]. Since most adults are sexually active, they may need an annual HIV screening if they have new partners or multiple partners, or if they deal with other risk factors, so lifetime screening is not enough. In particular, if deaf adolescents and adults have low HIV literacy, as Goldstein and others have reported [40-42], they may not request frequent screening based on their risk factors, leaving the responsibility to providers and outreach workers to educate and inquire about HIV screening frequency.

Specific demographic groups had disparate HIV screening rates in the past year in our deaf sample, with increased rates of recent HIV screening found among younger adults (18-34), nonCaucasian adults, and persons who identified as LGBQ. These results from our study were like other studies that identified MSM, Black, and younger adults as more likely to have received screening [9-12]. This suggests that hearing status or language use did not change the pattern of other sociodemographic factors contributing to HIV screening uptake likelihood. Male gender, as predictive in our sample, may reflect the overrepresentation of LGBQ and represent nonGBQ-identified MSM. It is less likely, yet possible, that our sample shows a recent upswing in HIV screening in heterosexual men after the 2015 CDC HIV screening recommendations, as seen following the 2006 recommendations [10]. The finding that deaf respondents with only high school education were less likely to ever have been screened is consistent with prior research that reported positive correlation between deaf ASL users’ health literacy and educational attainment [31]. If deaf adults are more aware of HIV risk factors, they may advocate for screening more often.

People screened were more likely to have had an STD in the past, which mirrors clinician recommendations for concurrent HIV counseling if presenting with an STD [2]. However, the HIV screening rates for both the past year and more than one year ago among respondents with an STD history were not different. The lack of an increased number of HIV screenings in the past year among respondents with a history of an STD may be a missed opportunity for increased frequency of screening and early HIV diagnosis, particularly among those who have a history of a bacterial STD [43,44].

Our modified Poisson prediction model showed a significant association of social media use with recent HIV screening, adjusting for sociodemographic correlates and STD history. Given that the adjusted prevalence ratio for HIV screening within the past year was more robust than the adjusted prevalence ratio for HIV screening over one year ago, social media may be particularly useful in enhancing frequent risk-based or exposure-driven screening rather than sustained routine screening habits. While this study did not interrogate causation, nor ask about respondents’ experiences with online or social media driven HIV screening campaigns, it is possible that the exchange of information through social media channels, including about HIV screening, sexual health, and PrEP, informs deaf individuals to seek HIV screening. We find it particularly interesting that social media use, as a main predictor, remained robust for recent screening considering the high prevalence of social media users in the past 12 months (91%). Such high utility provokes serious consideration as an avenue of outreach and shows its extensive reach across diverse deaf adults. Social media may be an avenue to mitigate cultural and linguistic barriers, particularly for deaf adolescents and young adults.

Evidence for social media and its role in promoting HIV-related awareness comes from a recent US study of 121 deaf GBQ men who used ASL, which found that a majority (85%) knew about PrEP for HIV prevention. Moreover, the perceived effectiveness of PrEP in preventing HIV was strongly associated with engaging in LGBTQ-related discussions online or on social networking sites [26]. While this study was restricted to GBQ men, and HIV screening knowledge and practice are not always equivocal, this offers further evidence that social media may play a powerful role in mitigating HIV screening disparities among deaf ASL users.

Strengths of this study include its diverse representation as a large population-based sample, including demographic and geographic variation across the United States, and its consistency with prior studies about HIV screening patterns in nonexclusively deaf populations. Conversely, our sample is not nationally representative, and some comparisons to other population-based estimates may be limited. Our study did not explore patient centered pretest counseling in which HIV screening was obtained, limiting our understanding of whether increased HIV screening uptake in our large deaf sample is synonymous with self-initiated requests for screening or self-assessments of HIV risk. Our interpretation of the survey data is limited in several key ways. The query about STD history was not defined and open to interpretation by each participant, which could extend to including HIV or excluding other lesser recognized STDs, potentially underestimating the number of participants with a history of STDs and its impact on HIV screening patterns. HIV status was not obtained, potentially affecting our prevalence of screening. Though given its overall low prevalence, we do not think obtaining HIV status would significantly impact our findings. The data is self-reported and subject to recall biases, which is typical for any survey in English or other languages.

### Conclusion

Given low to moderate screening rates, deaf patients should be evaluated whenever possible for the need for HIV screening and associated risk factors. As funding and resources for HIV or STD outreach programs continue to be tightened, program managers and healthcare providers should consider promoting culturally and linguistically accessible social media campaigns informing deaf ASL users of the need for routine HIV screening. Further research to better understand the implementation steps between education and screening uptake among deaf ASL users would inform potential interventions to reduce HIV-related disparities. Given potential gaps in health literacy, a logical next step might be mixed-methods interventional studies, using social media to enhance frequency of screening and self-assessment of risk factors. Expanded sexual behavior items among deaf ASL users would highlight groups that should be targeted by such social media outreach, regardless of screening prevalence. However, integration of social networking sites should take care to not overlook older adults or heterosexual adults who may not utilize social media as readily, requiring public health interventions to be multiplatform. A cohesive strategy, combined with increasing the evidence base, will mitigate existing gaps between subgroups and bring deaf adults closer to universal screening, diminishing the collective risk of HIV in this diverse and tightknit community.

## Abbreviations

aPR: adjusted prevalence ratios
ASL: American Sign Language
BRFSS: Behavioral Risk Factor Surveillance System
CDC: Centers for Disease Control and Prevention
GBQ: gay, bisexual, and queer
HINTS-ASL: Health Information National Trends Survey in American Sign Language
LGBQ: lesbian, gay, bisexual, and queer
LGBTQ: lesbian, gay, bisexual, transgender, and queer
MSM: men who have sex with men
NSSHB: National Survey of Sexual Health and Behavior
OR: odds ratio
PrEP: preexposure prophylaxis
STD: sexually transmitted disease
USPSTF: United States Preventive Services Task Force
